# Fatigue Behavior and Life Prediction of L-PBF Ti64 with Critical Plane Based Small Building Direction Variations Under Non-Proportional and Multiaxial Loading

**DOI:** 10.3390/ma18225122

**Published:** 2025-11-11

**Authors:** Tian-Hao Ma, Yu-Xin Wang, Le Chang, Wei Zhang, Jian-Ping Zhao, Chang-Yu Zhou

**Affiliations:** School of Mechanical and Power Engineering, Nanjing Tech University, Nanjing 211816, China

**Keywords:** multiaxial fatigue, laser powder bed fusion, Ti-6Al-4V, critical plane, artificial neural network

## Abstract

Multiaxial low-cycle fatigue (MLCF) behavior of laser powder bed fused (L-PBF) Ti-6Al-4V was systematically investigated with four building direction (BD) in this paper. Proportional and non-proportional strain-controlled MLCF tests characterized cyclic softening and fracture mechanisms. L-PBF Ti-6Al-4V exhibits three-stage cyclic softening with occasional initial hardening, while non-proportional softening predominates, contrasting with conventional titanium alloys. Macro-micro characterization reveals that defect density and cleavage morphology strongly influence fatigue performance across BD. Fatigue life was predicted using analytical models (FS and KBMP) and a hybrid physics- and data-driven VAE-ANN model. While the KBMP model improves predictions over FS, both fail to fully account for BD effects. Incorporating macro-micro features, the VAE-ANN model achieves highly accurate MLCF life predictions within 10% error. These results highlight the critical roles of BD and microstructural characteristics in governing the MLCF behavior of L-PBF Ti-6Al-4V.

## 1. Introduction

Additive Manufacturing (AM) has emerged as a transformative technology in advanced manufacturing, capable of overcoming the geometric limitations of conventional manufacturing (CM) through its layer-by-layer fabrication process [1,2]. Titanium and its alloys are widely employed in demanding industries such as aerospace and biomedical engineering due to their high specific strength, notable strain-hardening capacity, ductility, toughness, and excellent corrosion resistance [3,4,5]. Among AM titanium alloys, Ti-6Al-4V is the most extensively applied, primarily produced by Laser Powder Bed Fusion (L-PBF) and Laser Direct Energy Deposition (L-DED). The rapid solidification inherent to L-PBF typically results in a fine α′-martensitic microstructure, which enhances yield and ultimate strengths compared to L-DED and CM techniques like rolling or forging [6,7].

Fatigue remains a predominant failure mode in structural components. In AM materials, the layer-wise deposition process introduces microstructural anisotropy, with build orientation exerting a strong influence on fatigue resistance [8]. Furthermore, process-induced defects such as porosity and lack-of-fusion flaws can significantly degrade fatigue life [9,10]. Consequently, despite their favorable static properties, AM alloys often exhibit inferior fatigue performance relative to their CM counterparts, which constrains their broader adoption in engineering applications.

Several studies have investigated the effects of building direction (BD) and defects on the fatigue behavior of L-PBF Ti-6Al-4V. Nicoletto et al. [11] reported that specimens fabricated in vertical and horizontal orientations exhibited shorter fatigue lives, particularly in the vertical direction, due to poor surface finish. Fatemi et al. [12,13] emphasized that defects are not uniformly distributed across the cross-sections of as-built and machined AM specimens. Large lack-of-fusion defects are typically located near the surface but can be largely eliminated through machining, thereby improving fatigue performance even though smaller internal defects persist.

In fatigue behavior research, life prediction is of paramount importance for both CM and AM components. For AM alloys, life prediction is particularly challenging because fatigue resistance is influenced not only by applied loading but also by microstructural features and defect populations that are highly dependent on processing conditions [14]. Traditional fatigue life prediction methodologies can be broadly categorized into three types: equivalent, critical plane, and energy-based methods [15]. With advancements in data science, numerous data-driven approaches have been proposed, primarily falling into statistical methods and machine learning (ML)-based methods [16]. Zhou et al. [17] built a three-layer artificial neural network (ANN) model with knowledge-based features to predict MLCF life under irregular loading, validated using 304L stainless steel. Hornas et al. [18] utilized a framework based on ML and Spearman’s rank correlation analysis to examine the influence of different features on the fatigue life performance of AM Ti-6Al-4V. However, most models for lifetime prediction focus on univariate representations and struggle to capture the nonlinear relationships between fatigue life and its influencing parameters.

Despite these efforts, most studies on the fatigue behavior of L-PBF Ti-6Al-4V have been limited to a small number of fixed building directions. Given the proven efficacy of critical plane approaches in evaluating multiaxial fatigue for CM materials, applying such methods to AM alloys with varying BDs holds significant promise. To address this research gap, the present work systematically investigates the multiaxial low-cycle fatigue (MLCF) behavior of L-PBF Ti-6Al-4V fabricated with different building directions, analyzed through a critical-plane-based framework. Proportional and non-proportional strain-controlled MLCF tests were performed to characterize cyclic hardening/softening trends. Microstructural observations and fracture surface analyses were conducted using optical microscopy and scanning electron microscopy on as-received and failed specimens, respectively. Finally, fatigue life prediction models were developed and validated by combining analytical formulations with hybrid physics- and data-driven approaches, aiming to establish a reliable predictive framework for L-PBF Ti-6Al-4V under complex multiaxial loading conditions.

## 2. Materials and Experimental Procedures

Ti-6Al-4V powder supplied by EOS (EOS GmbH, Krailling, Germany), with a nominal particle size distribution of ~63 μm, was used in this study. The chemical composition complies with ASTM F1472 [19] and ASTM F2924 [20] standards. The fabrication was conducted on an EOS M290 L-PBF system employing processing parameters recommended by EOS: laser power of 280/150 W (infill/contour), scanning speed of 1200/1250 mm/s, hatch spacing of 140 μm, and layer thickness of 30 μm.

**Figure 1:**
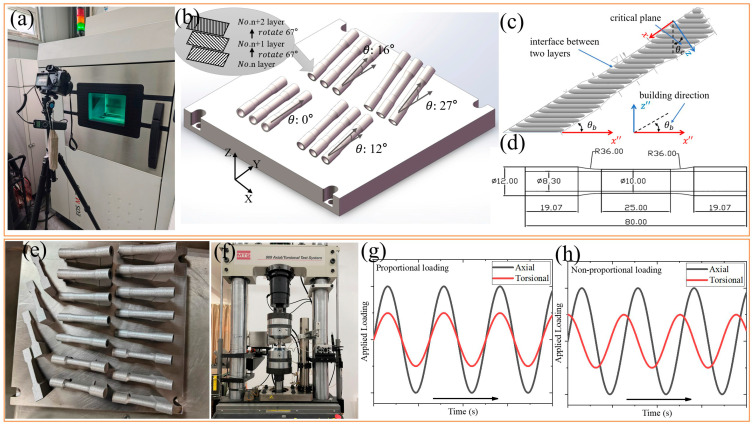
(**a**) EOS M290 L-PBF system, (**b**,**c**) building direction schematic, (**d**) specimen geometry, (**e**) as-received specimen, (**f**) MTS 809 material test system, (**g**) proportional loading path and (**h**) non-proportional loading path.

Specimens were produced in four building directions (0°, 12°, 16°, and 27°), illustrated in Figure 1a,b. The four building directions were selected based on our previous work [21], which applied critical plane theory to the MLCF behavior of a CM (rolled) titanium alloy. The critical plane approach is widely recognized in multiaxial fatigue research for its strong correlation with experimental observations of crack initiation and propagation [22,23]. Under multiaxial loading, fatigue cracks typically initiate and propagate along a specific angular plane, commonly referred to as the critical plane. In strain-controlled loading modes, the critical plane is typically defined as the plane of either maximum principal strain or maximum shear strain. In this study, the selected building directions are oriented at approximately 90° to the respective critical planes, as shown in Figure 1c. The mean fracture surface inclination and critical plane based on the maximum principal strain are 12.04°/16.88° with multiaxial strain ratio (λ) of 0.865 and 17.92°/26.58° with λ of 1.73, respectively [21]. Considering that the loading cases applied in this paper are almost based on λ of 0.865 and 1.73, the final selected building directions were 0°, 12°, 16° and 27°. The 0° direction was included as a baseline reference, commonly used in prior studies, to assess the effect of small-angle deviations aligned with critical planes on multiaxial fatigue behavior.

Following fabrication, specimens were detached from the baseplate using wire electrical discharge machining and subsequently heat-treated at 800 °C for 2 h in an argon atmosphere, as shown in Figure 1e. Subsequent lathe machining removed ~0.5 mm from both the inner and outer surfaces to eliminate major surface defects. The final specimen dimensions are provided in Figure 1d. To accommodate post-processing, the 3D “.STL” models were designed with dimensional allowances. A complete list of specimens and applied loading conditions used in this paper is given in Table 1.

MLCF tests were carried out on an MTS 809 servo-hydraulic system (MTS Systems Corporation, Eden Prairie, MN, USA) (Figure 1f) under both proportional and non-proportional strain-controlled modes at room temperature (20 °C, regulated by laboratory air conditioning). Sinusoidal waveforms were applied at a frequency of 1 Hz, with phase angles of 0° for proportional loading and 90° for non-proportional loading, as shown in Figure 1g and Figure 1h, respectively. Given the limitation, all analyses and conclusions in this paper are based on the aforementioned environmental temperature and applied strain. The repeatability of the fatigue life was validated using each three sets of duplicate specimens under both proportional and non-proportional loading paths. The observed repeated Nf values under the proportional loading path were: P02 vs. P02-R: 4752 vs. 5003, P06 vs. P06-R: 8982 vs. 8164 and P10 vs. P10-R: 1394 vs. 1176. The observed repeated Nf values under the non-proportional loading path were: NP02 vs. NP02-R: 7012 vs. 6458, NP06 vs. NP06-R: 6624 vs. 6093 and NP10 vs. NP10-R: 1013 vs. 1169. The deviation in fatigue life under identical loading conditions remains within 15%, confirming good experimental consistency.

**Figure 2:**
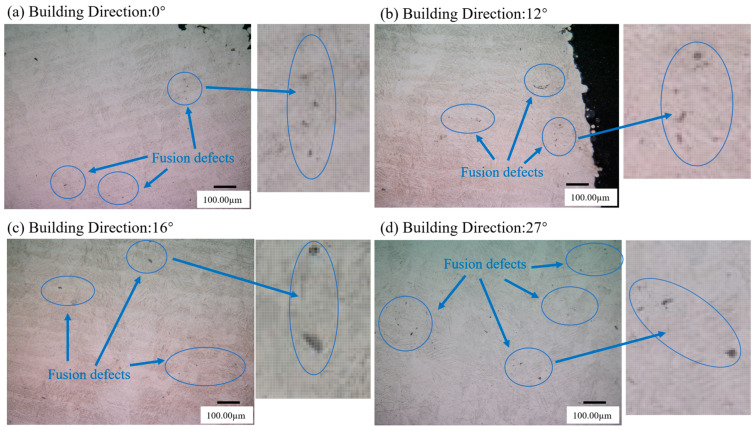
Metallography of as-received L-PBF Ti-6Al-4V with different building direction: (**a**) 0°, (**b**) 12°, (**c**) 16°, (**d**) 27°.

Metallographic examination was performed on cross-sections of as-received L-PBF Ti-6Al-4V specimens with four different BDs. The sections, prepared by wire cutting, were characterized using a VHX-950F digital microscope (Keyence, Osaka, Japan), as shown in Figure 2. The L-PBF Ti-6Al-4V exhibits additional fusion-related defects formed during the layer-by-layer deposition process. These defects, appearing as small voids, can be detected under optical microscopy (OM). Although the distribution of these Lack-of-Fusion (LOF) defects is highly random, they exert a pronounced influence on the material’s multiaxial fatigue behavior. Upon pixel-level magnification of the metallographic images, the contrast in LOF regions was significantly higher than in the surrounding material. Computer vision algorithms were employed to determine the proportional area of LOF defects based on these contrast differences. The quantified pixel-level density of LOF defects in as-received specimens for each building direction was subsequently utilized for the hybrid life prediction modeling in later sections.

## 3. Mechanical Behavior and Fatigue Fracture Mechanism of L-PBF Ti-6Al-4V with Different Building Directions

### 3.1. Static and Dynamic Mechanical Properties

Prior to the MLCF test, standard tensile and uniaxial low cycle fatigue (LCF) tests were conducted on specimens with different building directions to characterize the material’s basic static and dynamic mechanical properties. The tensile and uniaxial properties of L-PBF Ti-6Al-4V for the four BDs are summarized in Table 2.

Overall, the tensile strength and yield strength of L-PBF Ti-6Al-4V are slightly higher than those of rolled Ti-6Al-4V ($\sigma_{u}$ = 1050 MPa, $\sigma_{y}$ = 855 MPa), the elastic modulus is slightly higher or lower than that of rolled Ti-6Al-4V (E = 106.2 GPa) [24], varying with the building direction. The mean ratio for $\sigma_{u}/\sigma_{y}$ of L-PBF Ti-6Al-4V is 1.17, suggesting a tendency for cyclic softening [25]. To obtain the dynamic mechanical properties of L-PBF Ti-6Al-4V, the Ramberg–Osgood model [26] was developed to describe a material’s dynamic mechanical behavior for the elastic and plastic regions of the stress–strain curve, as shown in Equation (1). Established empirical rules for the Ramberg–Osgood parameters suggest that a material is prone to cyclic softening when $\sigma_{u}/\sigma_{y}$ < 1.2 and $n^{\prime }$ < 0.2 [27].

(1) $$\frac{∆\varepsilon }{2}=\frac{∆\sigma }{2E}+{\left(\frac{∆\sigma }{2K^{\prime }}\right)}^{\frac{1}{n^{\prime }}}\;$$

### 3.2. Cyclic Softening/Hardening Characteristics

**Figure 3:**
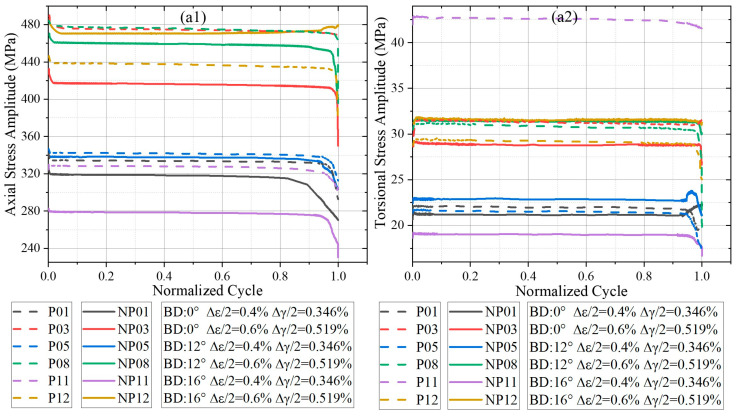

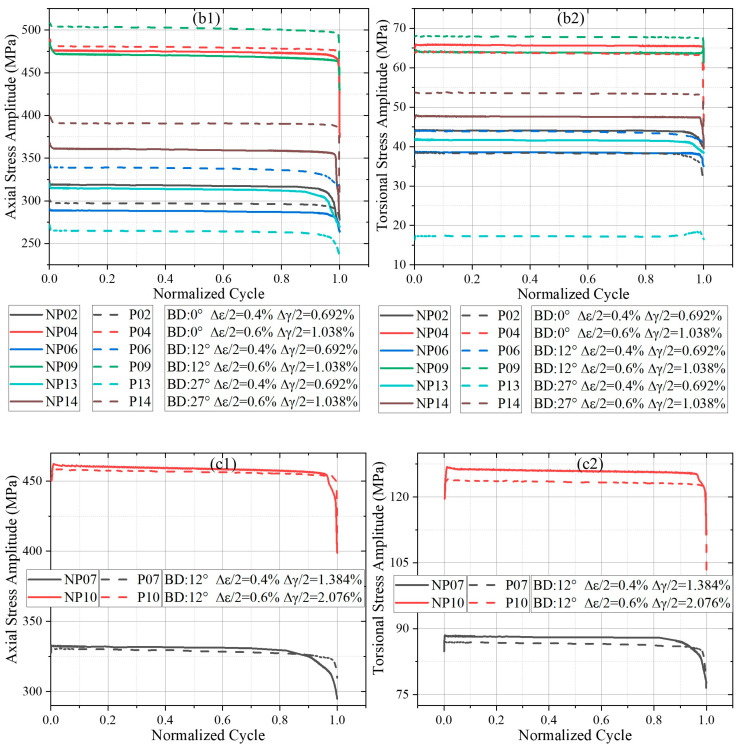
Stress amplitude response of L-PBF Ti-6Al-4V with different BDs: (**a1**) Axial under λ = 0.865, (**a2**) Torsional under λ = 0.865, (**b1**) Axial under λ = 1.73, (**b2**) Torsional under λ = 1.73, (**c1**) Axial under λ = 3.46, (**c2**) Torsional under λ = 3.46.

The cyclic stress response, which reflects the material’s softening or hardening behavior, was evaluated by tracking the evolution of stress amplitude with the number of cycles under proportional and non-proportional MLCF loading. Figure 3 presents the variations in axial and torsional stress amplitudes for different λ under proportional and non- proportional loading. To facilitate comparison across different conditions, the fatigue lives were normalized to the cycle fraction. For the axial response, specimens tested at λ = 0.865 and 1.73 exhibited a typical three-stage softening trend: continuous softening, a quasi-stable stage, and finally rapid softening. In contrast, specimens at λ = 3.46 exhibited a short initial hardening phase, followed by a softening process similar to the other cases. Under non-proportional loading, the differences in axial stress response between different build directions were more pronounced than those observed under proportional loading, indicating that the axial response of L-PBF Ti-6Al-4V is more sensitive to BD in non-proportional loading. In torsion, the absolute stress amplitudes were markedly lower than axial amplitudes across all conditions, and the differences between proportional and non-proportional loading were relatively small. This suggests that axial loading plays a dominant role in governing the stress response of L-PBF Ti-6Al-4V, regardless of λ or loading conditions. In Figure 3, proportional and non-proportional loading cases are distinguished by line style, with colors corresponding to the same loading level. In both axial and torsional responses, proportional loading consistently produced higher stress amplitudes than non-proportional loading. Thus, non-proportional hardening—commonly reported in conventionally manufactured titanium alloys—is essentially absent in L-PBF Ti-6Al-4V. Instead, a trend of non-proportional softening was observed, in contrast to the stabilization or hardening typically reported in wrought Ti alloys such as BT9 [28].

### 3.3. Fatigue Fracture Mechanism of L-PBF Ti-6Al-4V

**Figure 4:**
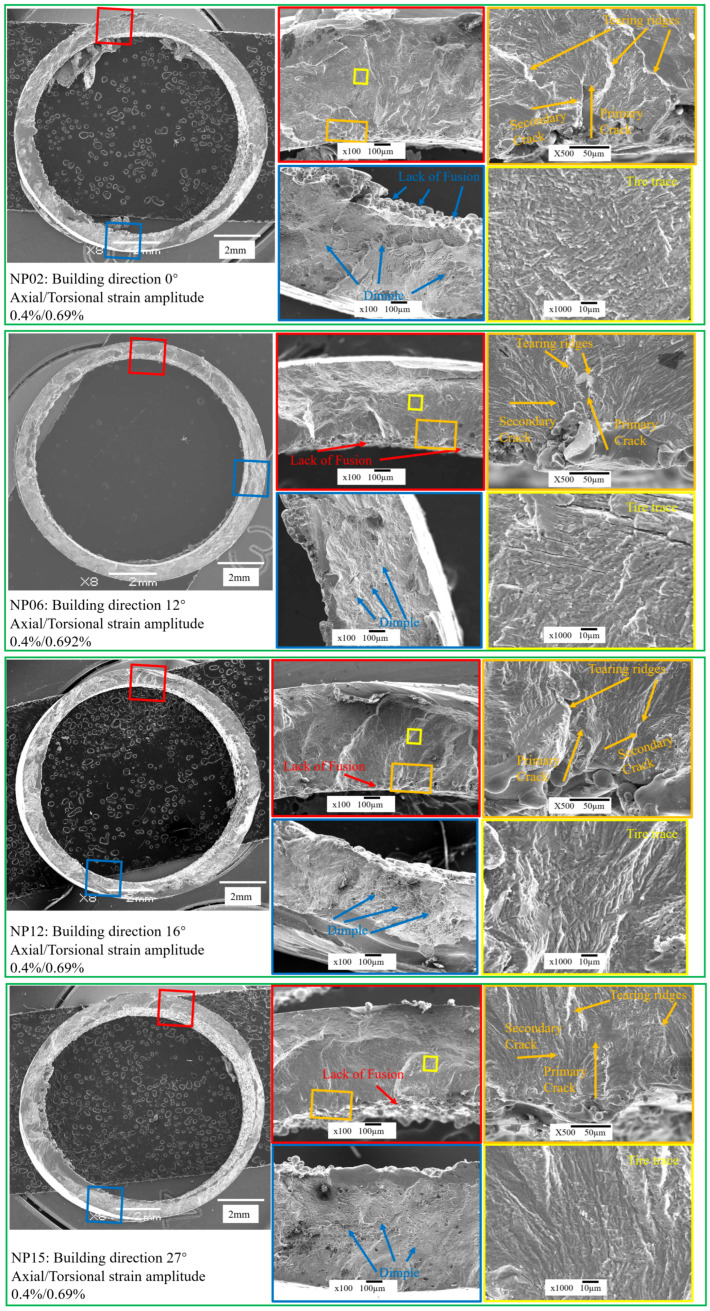
Fracture morphology of L-PBF Ti-6Al-4V.

To elucidate the fatigue crack initiation and propagation mechanisms of L-PBF Ti-6Al-4V with different BDs, post-MLCF fracture surfaces were analyzed using a JEOL JSM-7800F field emission gun Scanning Electron Microscopy (SEM) (JEOL Ltd., Akishima, Tokyo). Figure 4 displays the fracture surface morphology of representative specimens tested under non-proportional loading at an axial strain amplitude of 0.4%, highlighting the crack initiation region, crack propagation region, and final rupture region.

In specimens NP02, NP06, NP12, and NP15, small LOF defects were identified within the crack propagation zones. These LOF defects acted as preferential sites for fatigue crack initiation from the internal surface. Further magnification of the crack propagation zone reveals tearing ridges and fatigue striations which propagate along different directions (orange box). Primary crack growth occurred radially outward, accompanied by secondary circumferential propagation. Characteristic “tire trace”, typically associated with axial–torsional fatigue loading, were also observed in the crack propagation zone (yellow box).

Among the four BDs, NP06 exhibited the most pronounced river-pattern features, characterized by cleavage and quasi-cleavage facets. Cleavage, representing brittle transgranular fractures across well-defined crystallographic planes, is the most detrimental fracture mode, accounting for the shortest fatigue life of NP06 under identical loading conditions.

In general, a greater presence of cleavage planes indicates higher brittleness and reduced plasticity, both of which are unfavorable for fatigue performance. In contrast, the NP12 specimen, which achieved the longest fatigue life, displayed the fewest cleavage features. Its crack propagation zone was dominated by elongated tearing ridges and fatigue striations rather than cleavage planes. The variation in cleavage characteristics across different BDs demonstrates that BD significantly influences the plastic properties of L-PBF Ti-6Al-4V under multiaxial fatigue loading. These observations highlight the strong influence of BD on the balance between plastic and brittle behavior of L-PBF Ti-6Al-4V under multiaxial loading. Optimization of BD can therefore enhance plastic deformation capacity and improve fatigue resistance. The differences in plastic and brittle behavior under varying BDs are also reflected in the size and density distribution of dimples within the final rupture zone (blue box).

## 4. MLCF Life Prediction of L-PBF Ti-6Al-4V

### 4.1. Fatigue Life Distribution of L-PBF Ti-6Al-4V

**Figure 5:**
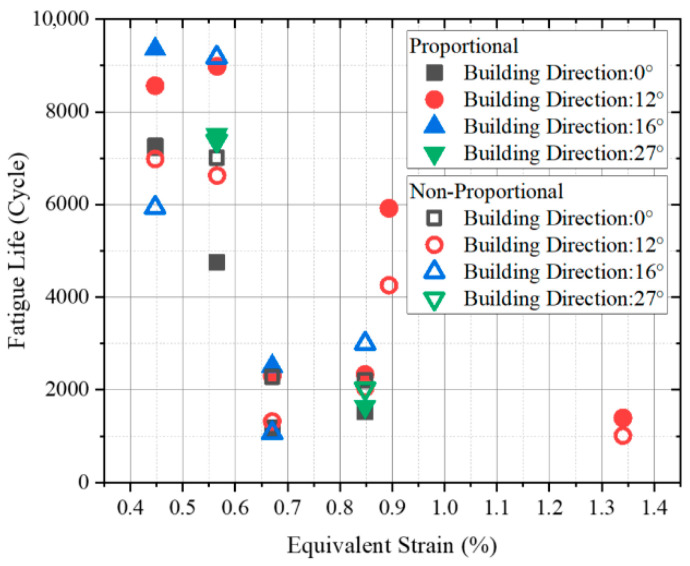
Fatigue life distribution of L-PBF Ti-6Al-4V with different building directions: equivalent strain—fatigue life.

Figure 5 illustrates the fatigue life distributions of the investigated specimens based on the equivalent strain. The von Mises equivalence criterion was applied under both axial and torsional loading, with the equivalent strain calculated using Equation (2) [29]:

(2) $$\varepsilon_{equiv}=\sqrt{{\left(∆\varepsilon /2\right)}^{2}+{\left(∆\gamma /2\right)}^{2}/3}$$

where ∆ε/2 and ∆γ/2 are the axial and torsional strain amplitude, respectively.

As shown in the fatigue life distribution in Figure 5, the MLCF life of L-PBF Ti-6Al-4V exhibits pronounced scatter even under identical equivalent strain levels, independent of BD and loading path (proportional or non-proportional). It is noteworthy that the dispersion is more pronounced under lower applied equivalent strain amplitude levels than under higher levels. This observed variability underscores the importance of either modeling different building directions and loading conditions individually or explicitly accounting for their effects within life prediction models.

### 4.2. MLCF Life Prediction Based on Analysis Formula Method

#### 4.2.1. Fatemi–Socie Model

The well-established Fatemi–Socie (FS) model was first employed to assess its predictive capability for L-PBF Ti-6Al-4V. Fatemi et al. [30] proposed this modification of the original KBM model [31], incorporating both strain and stress parameters to better describe shear-dominated failure, as given in Equation (3):

(3) $$\frac{∆\gamma_{max}}{2}\left(1+n\frac{\sigma_{n}^{max}}{\sigma_{y}}\right)=\left(1+v_{e}\right)\frac{\sigma_{f}^{\prime }}{E}{\left(2N_{f}\right)}^{b}+\frac{n}{2}\left(1+v_{e}\right)\frac{{\sigma_{f}^{\prime }}^{2}}{E\sigma_{y}}{\left(2N_{f}\right)}^{2b}+\;\left(1+v_{p}\right)\varepsilon_{f}^{\prime }{\left(2N_{f}\right)}^{c}+\frac{n}{2}\left(1+v_{p}\right)\frac{\varepsilon_{f}^{\prime }\sigma_{f}^{\prime }}{\sigma_{y}}{\left(2N_{f}\right)}^{b+c}$$

where $∆\gamma_{max}$, $\sigma_{n}^{max}$, and $\sigma_{y}$ are the maximum shear strain range and the maximum normal stress and yield stress in the maximum shear strain plane, which are listed in Table 3 respectively. Values of $v_{e}$ and $v_{p}$ are 0.34 and 0.5, respectively. n is a constant reflecting the effect of torsional loading. For simplification, the fatigue parameters $\sigma_{f}^{\prime }$, $\varepsilon_{f}^{\prime }$, b, and c in the FS model are aligned with uniaxial fatigue parameters of rolling CP-Ti. All values of fatigue parameters are summarized in Table 4. The fatigue lives predicted by the FS model are compared with experimental data in the following section. A comparative analysis of the predictive accuracy across different models will be conducted in Section 4.4.

#### 4.2.2. Proposed KBMP Model

Building on our earlier studies of commercially pure titanium [21,32], we modified the KBM model [33] to the proposed KBMP model, which better captures MLCF behavior and critical plane response of titanium, as shown in Equation (4):

(4) $$\frac{∆\gamma_{max}}{2}+S_{p}∆\varepsilon_{n}=\left[1+v_{e}+S_{p}\left(1-v_{e}\right)\right]\frac{\sigma_{f}^{\prime }}{E}{\left(2N_{f}\right)}^{b}+\left[1+v_{p}+S_{p}\left(1-v_{p}\right)\right]\varepsilon_{f}^{\prime }$$

where $∆\gamma_{max}$ and $∆\varepsilon_{n}$ are the maximum shear strain range and normal strain range on the maximum principal strain plane under strain-controlled mode, which are listed in Table 3 respectively. $v_{e}$ and $v_{p}$ are the elastic and plastic Poisson’s ratios, respectively. The values of $v_{e}$ and $v_{p}$ are 0.34 and 0.5, respectively [34]. The fatigue parameters $\sigma_{f}^{\prime }$, $\varepsilon_{f}^{\prime }$, b, and c in the KBMP model are identical to those adopted in the FS model. The parameter $S_{p}$ represents the influence coefficient of normal strain on the critical plane defined by the maximum principal strain. Complete parameter values are listed in Table 4. Predicted fatigue lives obtained from the KBMP model are compared with experimental results in the following section. A comparative analysis of the predictive accuracy across different models will be conducted in Section 4.4.

### 4.3. MLCF Life Prediction Based on Hybrid Physics and Data-Driven Method

**Figure 6:**
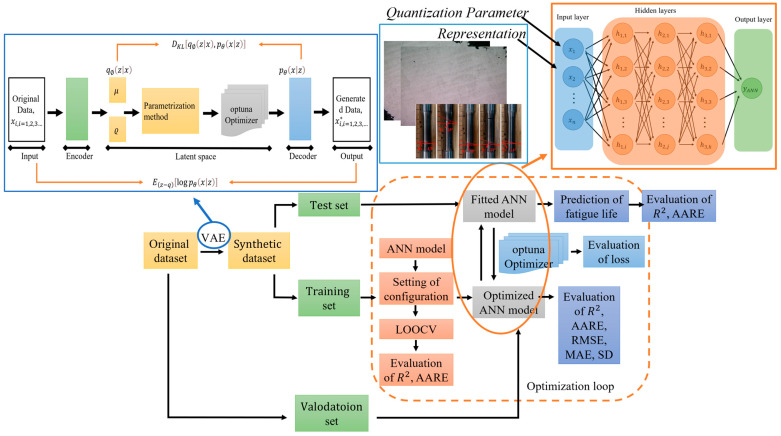
Workflow of the proposed VAE-ANN model.

With the advancement of data science, numerous fatigue life prediction strategies have been developed using machine learning (ML) techniques, such as the Artificial Neural Network (ANN) [35,36], Support Vector Machine (SVM) [37,38] and Gaussian Process (GP) [39]. Among these, ANN has been the most widely adopted and has demonstrated significant value in addressing fatigue-related problems [40]. However, purely data-driven ML models often encounter challenges such as overfitting and limited interpretability. To mitigate these limitations, hybrid physics–ML frameworks have increasingly emerged as the preferred approach [41]. Our previous research [42] developed a VAE-ANN model integrating data expansion with hybrid physical and data-driven information, enabling improved prediction of fatigue life under complex multiaxial loading. In the present study, we further extend this framework by incorporating macro– and microstructural characterization to account for the influence of building direction and loading mode on the MLCF behavior of L-PBF Ti-6Al-4V. The complete workflow of the proposed VAE-ANN model is illustrated in Figure 6.

#### 4.3.1. Influencing Parameters on MLCF Life of L-PBF Ti-6Al-4V

Based on the aforementioned MLCF models, the key parameters influencing the MLCF life of L-PBF Ti-6Al-4V can be categorized as follows: (1) Load parameters: The value of strain amplitude or stress amplitude directly reflects the level of loading. The phase angle, on the other hand, reflects the proportional or non-proportional loading path; (2) Critical plane parameters: The angle of critical plane can identify which plane is likely to experience the most extreme damage. The strain or stress coefficients on the critical plane can then account for damage on specific material planes. Those cases involving multiple out-of-phase load inputs can be treated with high accuracy; (3) L-PBF-based parameters: Building direction, crack propagation angle and LoF defects density reveals macro- and microstructural information about L-PBF Ti-6Al-4V.

However, many of these parameters require empirical calibration to predict the multiaxial fatigue life of different materials, and some involve time-consuming and costly experimental measurements. Leveraging the powerful feature extraction capabilities of ML and the availability of relevant test data from existing studies, a novel data-driven method is proposed. Twelve parameters which are relatively easy to measure or obtained are determined as the input features for the data-driven model, as shown in Table 3.

**Table 3:** The input features.

	Input Features	Symbol	Example
Load-based parameters	Axial and torsional strain amplitude (%)	∆ε/2, ∆γ/2	(0.4, …, 2.976)
	Axial and torsional stress amplitude (MPa)	∆σ/2, ∆τ/2	(30, …, 395)
	Phase angle (°)	ϕ	(0, …, 90)
Critical plane-based parameters	Maximum shear strain range (%) and Maximum normal stress (MPa) [30]	$∆\gamma_{max}$, $\sigma_{n}^{max}$	(0.26, …, 378.15)
	Maximum shear and Normal stress range (MPa) [43]	$∆\tau_{max}$, $∆\sigma_{n}$	(37.05, …, 703.77)
L-PBF-based parameters	Building Direction (°)	BD	(0, …, 27)
	Crack Propagation Angle (°)	CPA	(13, …, 29)
	Lack-of-Fusion Defects Density (Pixel/Pixel)	LOFD	(1 × 10^−4^, …, 1 × 10^−3^)

#### 4.3.2. Artificial Neural Network with Data Expansion

As a data-driven model, the predictive accuracy of an ANN is highly dependent on the size of the training dataset [44,45]. Applying the ANN model to fatigue life prediction with limited number of samples may lead to adverse results in that: (1) the relationship between the fatigue life and the influencing factors is formed directly from the training samples, which may yield results that contradict established fatigue mechanics principles; (2) insufficient sample diversity can cause overfitting, where the model fails to generalize well, particularly for extrapolative predictions beyond the range of the training data.

To mitigate the challenge of limited data availability for L-PBF Ti-6Al-4V under MLCF loading, a Variational Autoencoder (VAE) was integrated into the framework to augment the original dataset. The VAE is a stochastic generative model that learns the underlying probability distribution of a given dataset and can generate new, synthetic data samples [46]. VAE model consists of an encoder and a decoder. The encoder uses the inference network to generate the variational probability distribution $q_{\emptyset }(z\left|x\right.)$ of the hidden variable z from the original input data X. The decoder uses the generation network to restore the probability distribution $p_{\theta }\left(x\left|z\right.\right)$ of generated data $X^{*}$ according to $q_{\emptyset }\left(z\left|x\right.\right)$. The input to VAE is the original data $X_{i,i=1.2.3\ldots }$, and the output is the generated data ${X^{*}}_{i,i=1.2.3\ldots }$. Nonlinear activation functions are employed in both the encoder and decoder to capture complex relationships between the input and output variables. Leaky ReLU function is used as the activation function comparison group for the latent space in the VAE model, as shown in Equation (5):

(5) $$f_{Leaky\;ReLU}\left(x\right)=\left\{\begin{array}{c} x\;\;\;\;\;\;\;\;\;\;\;\;\;\;if\;x>0\\ 0.01x\;\;\;\;\;\;\;\;otherwise \end{array}\right.$$

This design enables the VAE to generate realistic synthetic data that reflects the statistical characteristics of the original MLCF dataset, thereby enhancing the training set for subsequent ANN modeling. ANN is a widely recognized machine learning algorithm, particularly effective for capturing complex nonlinear relationships. The architecture of ANN is inspired by the structure and functionality of biological neural networks. In this study, a five-layer perceptron ANN regressor is employed. Within the hidden layers, each neuron transforms the outputs from the previous layer through a linear weighted summation followed by a nonlinear activation function $f_{j}$, the output of the node is given in Equation (6):

(6) $$y_{i}=f_{j}(b_{j}+\sum_{i=1}^{d}\omega_{i,j}x_{i})$$

where $\omega_{i,j}$ is the weight of neural network, $x_{i}$ represents the input of current neuron node, and $f_{j}$ using Leaky ReLU activation function.

The dataset is partitioned into training and testing sets with a split of 69% and 31%, respectively. Input features are grouped into three categories: fatigue parameters derived from analytical formulations, loading conditions, and microstructural characteristics. Macro-micro information consists of the initial defect density per unit area in the as-received state and the crack surface inclination angle after fracture. The output parameter is fatigue life. To facilitate stable optimization and enhance the model’s predictive accuracy, both input and output features are normalized to the range [0, 1] according to Equation (7) [47]:

(7) $$x^{*}=\left\{\begin{array}{c} \frac{x-x_{min}}{x_{max}-x_{min}}\;\;\;\;\;\;\;\;\;\;\;\;\;\;\;\;\;\;\;\;\;\;\;\;\;\;\;\;\;\;\;\;\;\;\;\;\;\;\;\;\;\;\;\;\;\;x\;is\;an\;input\;feature\\ \frac{\log_{10}x-\log_{10}x_{min}}{\log_{10}x_{max}-\log_{10}x_{min}}\;\;\;\;\;\;\;\;\;\;\;\;\;\;\;\;\;\;\;\;\;\;\;\;x\;is\;an\;output\;feature \end{array}\right.$$

**Figure 7:**
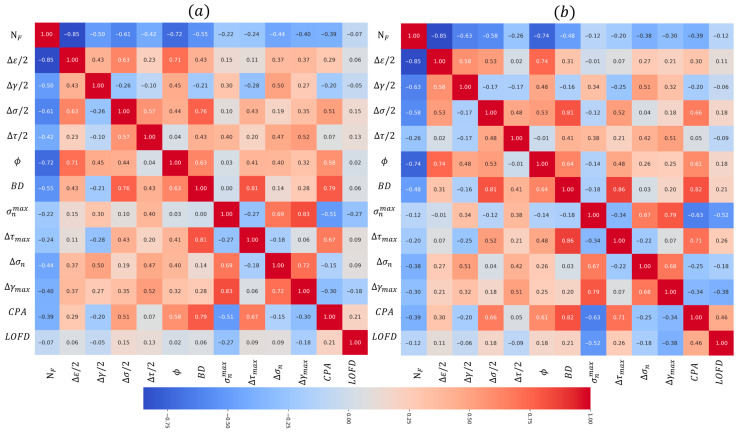
Pearson correlation coefficients for input features of: (**a**) test data, (**b**) extended data.

To further investigate the correlation among input features, the Pearson Correlation Coefficient (PCC) is applied to test dataset and extended dataset, as shown in Equation (8) and Figure 7.

(8) $$\eta (PCC\left(X,Y\right))=\frac{\sum_{i=1}^{n}\left(X_{i}-\bar{X})(Y_{i}-\bar{Y}\right)}{\sqrt{\sum_{i=1}^{n}{\left(X_{i}-\bar{X}\right)}^{2}}\sqrt{\sum_{i=1}^{n}{\left(Y_{i}-\bar{Y}\right)}^{2}}}$$

The absolute value of the Pearson Correlation Coefficient (PCC) reflects the strength of the linear relationship between two features, with values closer to 1 indicating stronger correlations. A positive PCC denotes a positive correlation, while a negative value signifies a negative correlation. As the PCC matrix is symmetric about the diagonal, the corresponding labels in the figure are also symmetrically arranged. From Figure 7a, it can be seen that only the PCC values between $∆\tau_{max}$ and BD, $∆\gamma_{max}$ and $\sigma_{n}^{max}$ of the original test dataset above 0.8. This PCC analysis suggests that the selected input information—encompassing load-based, critical plane-based and L-PBF-based parameters—possess reasonable redundancy, thereby indicating a low risk of overfitting. When compared with Figure 7a, the PCC distribution of the expanded dataset in Figure 7b closely matches that of the original test data. The high consistency between the two PCC matrices confirms the reliability of the data expansion method.

#### 4.3.3. Hyperparameter Optimization

In this study, hyperparameter optimization was conducted using the Covariance Matrix Adaptation Evolution Strategy (CMA-ES). CMA-ES is a powerful evolutionary algorithm that iteratively adapts the search distribution based on previously sampled solutions [48]. The adaptation process involves updating not only the mean (*μ*) and step-size (ϱ), but also the full covariance matrix of the parameter distribution.

At each generation, CMA-ES generates parameters from a multi-variate normal distribution to sample solutions. It focuses on the best $N_{best}$ solutions in the current generation, with $N_{best}$ set to the best 25% of solutions. The mean $\mu^{\left(g+1\right)}$ for the next generation is then calculated based on these best solutions, as shown in Equations (9) and (10):

(9) $$\mu_{x}^{\left(g+1\right)}=\frac{1}{N_{best}}\sum_{i=1}^{N_{best}}x_{i}$$

(10) $$\mu_{y}^{\left(g+1\right)}=\frac{1}{N_{best}}\sum_{i=1}^{N_{best}}y_{i}$$

Only the best 25% of the solutions are used to estimate the covariance matrix $C^{\left(g+1\right)}$ for the next generation, as shown in Equations (10)–(13):

(11) $$\varrho_{x}^{2,(g+1)}=\frac{1}{N_{best}}\sum_{i=1}^{N_{best}}{\left(x_{i}-\mu_{x}^{\left(g\right)}\right)}^{2}$$

(12) $$\varrho_{y}^{2,(g+1)}=\frac{1}{N_{best}}\sum_{i=1}^{N_{best}}{\left(y_{i}-\mu_{y}^{\left(g\right)}\right)}^{2}$$

(13) $$\varrho_{xy}^{\left(g+1\right)}=\frac{1}{N_{best}}\sum_{i=1}^{N_{best}}\left(x_{i}-\mu_{x}^{\left(g\right)})(y_{i}-\mu_{y}^{\left(g\right)}\right)$$

The stochastic gradient descent algorithm is employed for training by minimizing the loss function. For neuron *j* in the *l*-th layer, the adaptations of the *i*-th connection weight and bias are expressed as Equations (14) and (15).

(14) $$∆\omega_{ij}^{\left(l\right)}\left(t+1\right)=-\eta \cdot E\frac{\partial E}{\partial \omega_{ij}^{\left(l\right)}}+\theta \cdot ∆\omega_{ij}^{\left(l\right)}\left(t\right)$$

(15) $$∆b_{j}^{\left(l\right)}\left(t+1\right)=-\eta \cdot E\frac{\partial E}{\partial b_{j}^{\left(l\right)}}+\theta \cdot ∆b_{j}^{\left(l\right)}\left(t\right)$$

During the hyperparameter optimization process, the optimal configuration for both the VAE and the integrated VAE-ANN model was identified. Hyperparameters are explicitly restricted parameters that affect the training process. The hyperparameters of the optimized VAE model (latent_dim, learning_rate, batch_size) and VAE-ANN model (hidden_dim1, hidden_dim2, hidden_dim3, learning_rate) are listed in Table 4. For comparison, Table 4 also includes the fatigue parameters of FS model and KBMP model, both of which are based on analytical formulations. To examine whether increased model complexity leads to improved predictive performance, two different parameter scales (Model 1 and Model 2) were evaluated for the VAE-ANN architecture.

**Table 4:** Values of various fatigue parameters for different models.

λ	Phase Angle	FS/n	KBMP/$S_{p}$	Phase Angle	FS/n	KBMP/$S_{p}$
0.865	0	−0.057	1.30	90	−0.054	0.94
1.73		−0.065	1.36		−0.069	1.73
3.46		−0.084	1.21		−0.071	1.62
Data Expansion Model	Latent_dim	3	Learning_rate	5.06 × 10^−3^	Batch_size	28
	Hidden_dim1	Hidden_dim2	Hidden_dim3	Learning_rate	Training Time per Epoch	Epoch at Convergence
Model1	169	128	54	7.653 × 10^−4^	11 s	185
Model2	442	153	75	3.713 × 10^−3^	24 s	156

**Figure 8:**
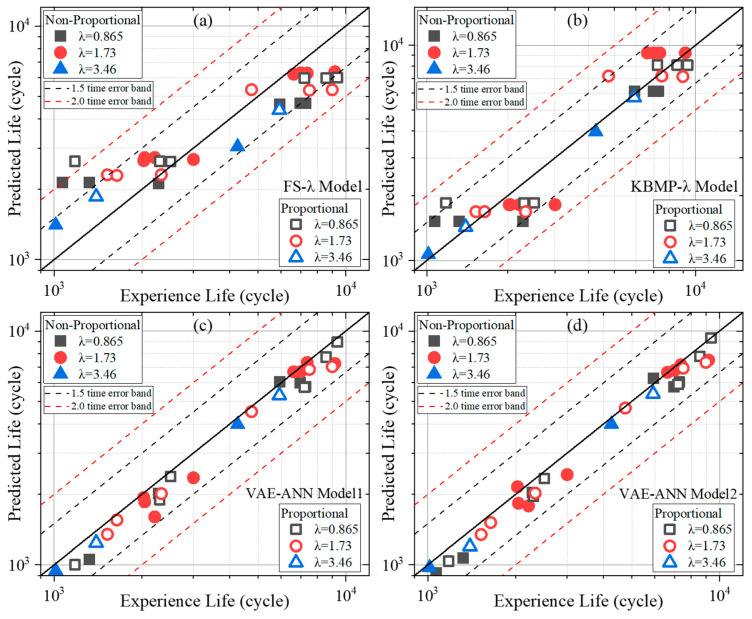
Comparisons between experimental life and predicted life predicted by (**a**) FS model, (**b**) KBMP model, (**c**) VAE-ANN model1, (**d**) VAE-ANN model2.

The fatigue lives predicted by the two VAE-ANN models are compared with experimental data in Figure 8c,d. The training time per epoch and the number of epochs required for convergence were 11 s and 185 epochs for VAE-ANN Model 1, and 24 s and 156 epochs for Model 2, respectively. The loss functions for both the training and test sets at the epoch of convergence remained below 0.001. Although the expanded parameter range in Model 2 accelerated convergence, it also resulted in a longer training time per epoch. Consequently, VAE-ANN Model 1 maintained a lower total training time overall.

### 4.4. Comparison of Life Prediction Performance of Different Models

As shown in Figure 8a,b, the predictions of the KBMP model generally fall within a 1.5-factor error band, whereas those of the FS model occasionally exceed the 2-factor error band. This indicates that the KBMP model delivers superior predictive accuracy between the two analytical models. However, both models exhibit a distinct cluster of data points along horizontal lines, implying that identical predictions are generated for specimens with different actual fatigue lives. This limitation arises from the inability of formula-based models to effectively incorporate the influence of BD into their framework. Attempting to re-fit separate parameter sets for each BD would lead to overly restrictive models with an inflated number of parameters, which is impractical for practical applications.

In contrast, the prediction results from the VAE-ANN models, presented in Figure 8c,d, show not only a significantly narrower error band but also the absence of such horizontal clustering. By incorporating macro- and microstructural characterization as input features, the hybrid physics- and data-driven VAE-ANN model can differentiate specimens with distinct BDs, enabling highly accurate fatigue life predictions. Furthermore, comparing the two VAE-ANN models with different parameter scales reveals that the prediction results are largely comparable. This suggests that increasing the parameter scale does not lead to a significant improvement in predictive performance but does incur a substantial increase in computational cost in this study.

In addition to the qualitative analysis of the life distribution predicted by each model, quantitative analysis is indispensable. Metrics including R-Squared ($R^{2}$), Average Absolute Relative Error (*AARE*), Root Mean Squared Error (*RMSE*), Mean Absolute Error (*MAE*) and Standard Deviation (*SD*) are employed to evaluate the predictive performance of each model, as defined in Equations (16)–(19).

(16) $$AARE=\frac{1}{n}\sum_{i=1}^{n}\left|\frac{\log_{10}N_{pi}-\log_{10}N_{fi}}{\log_{10}N_{fi}}\right|$$

(17) $$RMSE=\sqrt{\frac{1}{N}\sum_{i=1}^{N}{\left(\log N_{fi}-\log N_{pi}\right)}^{2}}$$

(18) $$MAE=\frac{1}{N}\sum_{i=1}^{N}\left|\log N_{fi}-\log N_{pi}\right|$$

(19) $$SD=\sqrt{\sum_{i=1}^{n}\frac{1}{n-1}{\left(\left|\frac{\log_{10}N_{pi}-\log_{10}N_{fi}}{\log_{10}N_{fi}}\right|-AARE\right)}^{2}}$$

**Figure 9:**
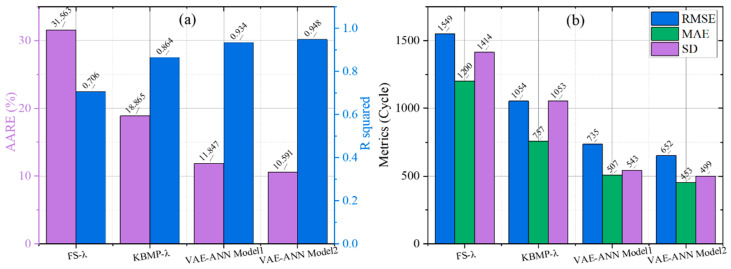
Quantitative error results of all models: (**a**) relative errors, (**b**) absolute errors.

The distribution of relative and absolute errors for each quantified model aligns with that shown in Figure 9. All error metrics for the KBMP model are higher than those of the FS model but lower than those of the VAE-ANN model. The two VAE-ANN models with different parameter scales exhibit essentially consistent quantitative errors. Among the formula-based models, the KBMP model achieves prediction accuracy within approximately 20% error, whereas the VAE-ANN model, combining both physics-based and data-driven approaches, further improves predictive accuracy to within 10%.

## 5. Conclusions

This study systematically examined the multiaxial fatigue behavior of L-PBF Ti-6Al-4V with different building directions under proportional and non-proportional loading. Cyclic stress response, fracture mechanisms, and fatigue life prediction were analyzed to elucidate the influence of building direction and microstructural features. The main conclusions are:

(1) L-PBF Ti-6Al-4V exhibits three-stage cyclic softening with occasional initial hardening. Under non-proportional loading, L-PBF Ti-6Al-4V exhibits non-proportional softening, in contrast to the non-proportional hardening typically observed in conventionally manufactured titanium alloys.
(2) Analytical fatigue life prediction models can provide reasonable estimates for L-PBF Ti-6Al-4V within 20% error band. The proposed KBMP model demonstrates superior performance over the traditional FS model.
(3) The hybrid VAE-ANN model, integrating physics-based parameters and macro-micro characterization, predicts MLCF life across different BDs with high accuracy within 10% error band and eliminates the horizontal data point limitations observed in formula-based models.

## Figures and Tables

**Table 1:** MLCF test data for L-PBF Ti-6Al-4V.

BD [°]	Strain Amplitude [%]		λ	Phase Angle [°]	Specimen No.	Nf [Cycle]	Phase Angle [°]	Specimen No.	Nf [Cycle]
	Axial	Torsional							
0	0.4	0.346	0.865	90	NP01	7272	0	P01	7217
0	0.4	0.692	1.73	90	NP02	7012	0	P02	4752
0	0.6	0.519	0.865	90	NP03	2280	0	P03	1178
0	0.6	1.038	1.73	90	NP04	2212	0	P04	1523
12	0.4	0.346	0.865	90	NP05	6980	0	P05	8562
12	0.4	0.692	1.73	90	NP06	6624	0	P06	8982
12	0.4	1.384	3.46	90	NP07	4256	0	P07	5918
12	0.6	0.519	0.865	90	NP08	1320	0	P08	2301
12	0.6	1.038	1.73	90	NP09	2044	0	P09	2332
12	0.6	2.076	3.46	90	NP10	1013	0	P10	1394
16	0.4	0.346	0.865	90	NP11	5935	0	P11	9352
16	0.6	0.519	0.865	90	NP12	1069	0	P12	2510
27	0.4	0.692	1.73	90	NP13	7375	0	P13	7503
27	0.6	1.038	1.73	90	NP14	2028	0	P14	1641
0	0.4	0.692	1.73	90	NP02-R	6458	0	P02-R	5003
12	0.4	0.692	1.73	90	NP06-R	6093	0	P06-R	8164
12	0.6	2.076	3.46	90	NP10-R	1169	0	P10-R	1176

**Table 2:** Tensile and Uniaxial Properties of L-PBF Ti-6Al-4V.

BD [°]	Static Mechanical Properties				Dynamic Mechanical Properties	
	Tensile Strength/$\sigma_{u}$ [MPa]	Yield Strength/$\sigma_{y}$ [MPa]	$\sigma_{u}/\sigma_{y}$	Elastic Modulus [GPa]	R-O Parameter/ $n^{\prime }$	R-O Parameter/ $K^{\prime }$
0	1266.76	1102.18	1.150	103.93	0.188	1003.91
12	1257.28	1062.41	1.184	108.01	0.176	926.50
16	1172.55	1000.63	1.171	108.03	0.179	971.63
27	1197.34	1032.35	1.160	102.38	0.181	963.84

## Data Availability

The original contributions presented in this study are included in the article. Further inquiries can be directed to the corresponding author.

## Abbreviations

The following abbreviations are used in this manuscript:

AARE	Average Absolute Relative Error
AM	Additive Manufacturing
ANN	Artificial Neural Network
BD	Building Direction
CM	Conventional Manufacturing
CMA-ES	Covariance Matrix Adaptation Evolution Strategy
CPA	Crack Propagation Angle
FS	Fatemi–Socie
GP	Gaussian Process
LCF	Low Cycle Fatigue
L-DED	Laser Direct Energy Deposition
LOF	Lack-of-Fusion
LOFD	Lack-of-Fusion Defects Density
L-PBF	Laser Powder Bed Fusion
MAE	Mean Absolute Error
ML	Machine Learning
MLCF	Multiaxial Low-Cycle Fatigue
OM	Optical Microscopy
PCC	Pearson Correlation Coefficient
RMSE	Root Mean Squared Error
SD	Standard Deviation
SEM	Scanning Electron Microscopy
SVM	Support Vector Machine
VAE	Variational Autoencoder
